# Survivin co-ordinates formation of follicular T-cells acting in synergy with Bcl-6

**DOI:** 10.18632/oncotarget.4994

**Published:** 2015-08-12

**Authors:** Karin M.E. Andersson, Mikael Brisslert, Nicola Filluelo Cavallini, Mattias N.D. Svensson, Amanda Welin, Malin C. Erlandsson, Michael J. Ciesielski, Gergely Katona, Maria I. Bokarewa

**Affiliations:** ^1^ Department of Rheumatology and Inflammation Research, The Sahlgrenska Academy at University of Gothenburg, Gothenburg, Sweden; ^2^ Department of Neurosurgery, Roswell Park Cancer Institute and State University of New York School of Medicine and Biomedical Sciences, Buffalo, NY, USA; ^3^ Department of Chemistry and Molecular Biology, University of Gothenburg, Gothenburg, Sweden; ^4^ Division of Cellular Biology, La Jolla Institute for Allergy & Immunology, La Jolla, CA, USA

**Keywords:** Immune response, Immunity, Immunology and Microbiology Section, survivin, Bcl6, T-cells, autoimmunity, arthritis

## Abstract

Follicular T helper (Tfh) cells are recognized by the expression of CXCR5 and the transcriptional regulator Bcl-6. Tfh cells control B cell maturation and antibody production, and if deregulated, may lead to autoimmunity. Here, we study the role of the proto-oncogene survivin in the formation of Tfh cells. We show that blood Tfh cells of patients with the autoimmune condition rheumatoid arthritis, have intracellular expression of survivin. Survivin was co-localized with Bcl-6 in the nuclei of CXCR5^+^CD4 lymphocytes and was immunoprecipitated with the Bcl-6 responsive element of the target genes. Inhibition of survivin in arthritic mice led to the reduction of CXCR5^+^ Tfh cells and to low production of autoantibodies. Exposure to survivin activated STAT3 and induced enrichment of PD-1^+^Bcl-6^+^ subset within Tfh cells. Collectively, our study demonstrates that survivin belongs to the Tfh cell phenotype and ensures their optimal function by regulating transcriptional activity of Bcl-6.

## INTRODUCTION

Follicular T helper cells (Tfh) is a subtype of memory T cells residing in secondary lymphoid structures, germinal centers (GC), and regulating B cell maturation and antibody production. Tfh cells exhibit specific phenotype by up-regulating chemokine receptor CXCR5 compulsory for their follicular localization, and surface molecules ICOS, PD-1, and SLAM, essential for interaction with B-cells [1, 2]. Generation and function of Tfh cells is a topic of special interest since their insufficiency is associated with immunodeficiency, while over-activation predispose to autoimmunity. The inflammatory milieu promotes the Tfh phenotype contributing with cytokines to activate the signal transducer and activator of transcription 3 (STAT3) [3, 4]. In particular, the cytokines IL-6, IL-27 and IL-21 have key roles at different stages of Tfh development and mediate STAT-3 dependent production of the transcriptional factor B-cell lymphoma 6 (Bcl-6) [5] [6, 7]. The expression of Bcl-6 in Tfh is prerequisite of GC formation and of extra follicular antibody responses [8]. In human setting, Tfh cell phenotype may also be induced through an alternative activation of IL-12 receptor and tyrosine kinase Tyk [9].

Rheumatoid arthritis (RA) is a chronic joint inflammation [10, 11]. The formation of ectopic GC is morphological characteristic feature of the inflamed rheumatoid synovium [12]. Synovial GCs are currently viewed as a place of affinity maturation and clonal expansion of B cells producing arthritis specific autoantibodies [13, 14]. The production of autoantibodies, which recognize the Fc-portion of human IgG, called rheumatoid factor (RF), and citrullinated protein epitopes (ACPA), is widely used in diagnostic of RA and also to identify the patients at risk to develop aggressive and therapy resistant RA [15–18]. It has recently been demonstrated that human circulating CXCR5+ memory T-cells are counterparts of the Tfh cells residing in GC and support differentiation of naïve B cells into plasmablasts and antibody producing plasma cells in healthy subjects and in autoimmunity [19–21]. The excess of Tfh cells in peripheral blood is reported in myasthenia gravis, thyroiditis, systemic lupus erythematosus, Sjögren's syndrome and juvenile dermatomyositis [2, 22]. Most of the reports show a correlation between Tfh cells and humoral autoimmunity in these patients, where the increased number of circulating Tfh is associated with disease activity, high levels of IL-21 and the presence of ACPA and RF [23–26]. Functionally active Tfh cells are identified in blood circulation during RA remission [27, 28] Notably, RA patients are early recognized by IL-6 dependent activation of STAT3 [29–32], the major driving mechanism of Tfh development. However, the origin of Tfh and molecular forces supporting their persistence in RA remains unrevealed.

This study addresses the role of the oncoprotein survivin in the formation of Tfh in RA. Following the initial description of survivin [33], the studies on survivin are concentrated on its anti-apoptotic and cell cycle regulatory properties in malignancies [34]. Later on, survivin was found important for the formation of functional TCR in the developing thymocytes [35, 36] and for the Flt3-dependent maturation of dendritic cells [37, 38]. In the immune competent cells, survivin production is required for the antigen presentation and for the formation of memory immune responses [39]. These processes are known as a hallmark of aberrant immunity in RA. In the established RA, high serum levels of survivin are frequently measured in patients producing RF and/or ACPA [40, 41]. The combination of survivin and autoantibodies recognizes RA patients with persistent disease activity and progressive joint damage [42, 43]. In the rheumatoid synovia, survivin was found in the areas enriched with macrophages and memory T cells [44–46]. Inhibiting survivin in arthritic mice restored the control over T-cell proliferation, and reduced arthritis [47]. At early pre-clinical stage of RA, serum levels of survivin correlated with the cytokines predisposing and assuring formation of Th1 and Th17 cells regulating the initial stages of RA pathology [48].

In this study we demonstrate that survivin belongs to the Tfh phenotype. It is expressed in the majority of circulating CXCR5+ memory T cells of RA patients and in arthritic mice. Changes in survivin transcription modulate formation and function of Tfh by regulating transcriptional activity of Bcl-6. This reduces CXCR5 expression and autoantibody production. Exposure to extracellular survivin restrains Tfh cells inducing PD-1^+^ Bcl-6^+^ phenotype of existing CXCR5^+^ survivin^+^ T cells.

## RESULTS

### Survivin and Bcl-6 are present in the peripheral blood follicular T helper cells of patients with rheumatoid arthritis

**Figure 1:**
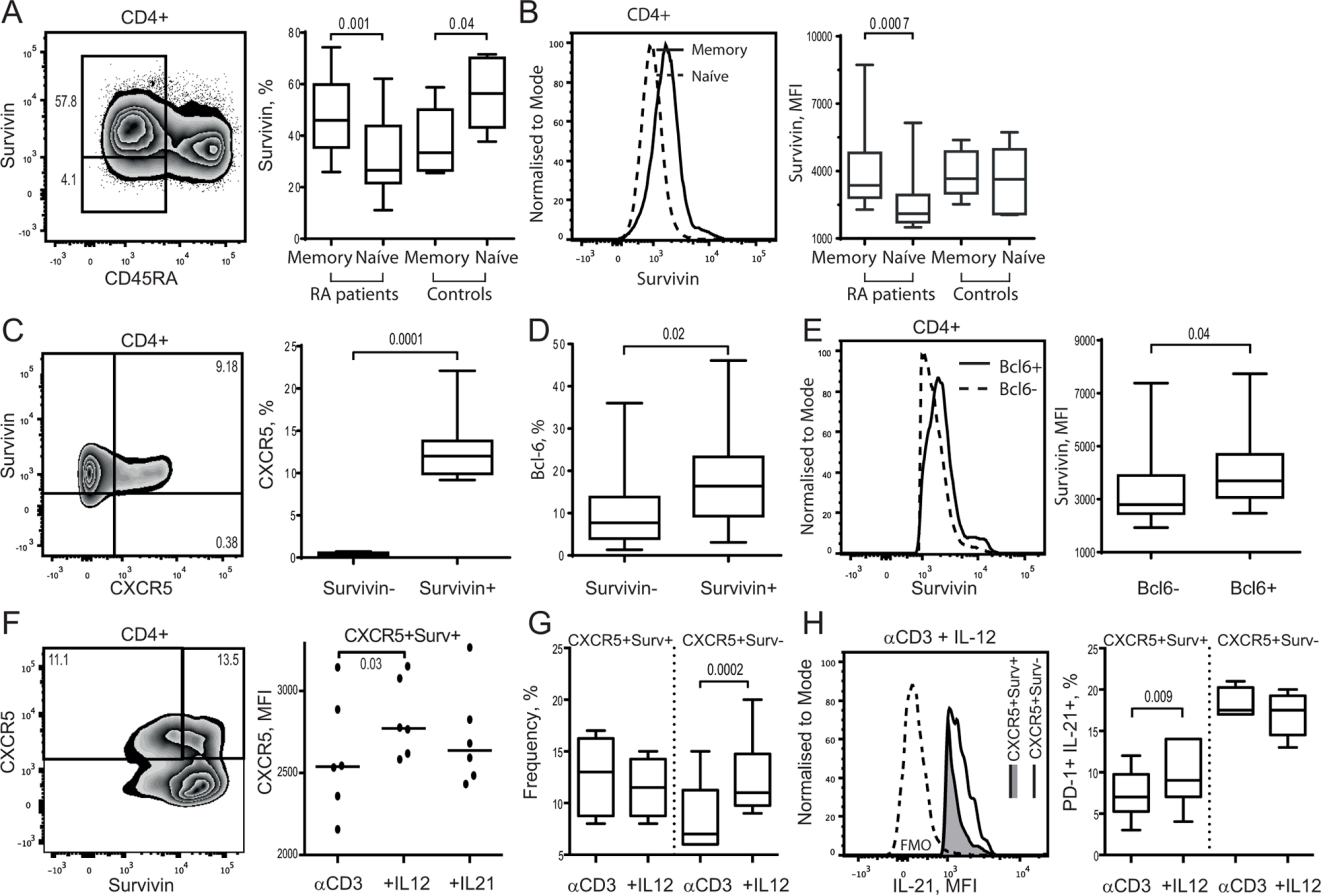
Survivin expression is an essential feature of human CXCR5^+^ Tfh cell phenotype Intracellular expression of survivin was investigated in memory (CD45RA^−^) or naïve (CD45RA^+^) CD4^+^ T cells of RA patients (*n* = 21) and healthy controls (*n* = 10) using flow cytometry. Cells are gated on CD4^+^ lymphocytes. Box plots show the frequency of survivin^+^ cells **A.** and the mean fluorescence intensity (MFI) of survivin **B.** Expression of CXCR5 **C.** within survivin^+^ and survivin^−^ CD4^+^ cells, and Bcl-6 **D.** within survivin^+^ and survivin^−^ memory (CD45RA^−^) CD4^+^ cells of RA patients. The intensity of survivin expression **E.** within Bcl-6^+^ and Bcl-6^−^ survivin^+^ CXCR5^+^ CD4 cells. The Mann-Whitney *U*-test was used to compare differences between groups. PBMCs of healthy subjects (1 × 10^6^/ml, *n* = 6) were cultured with anti-CD3 (0.25 μg/ml) alone or in combination with IL-12 (20 ng/ml) or IL-21 (50 ng/ml). On day 5, the formation of Tfh cells was recognized by expression of CXCR5 and intracellular production of IL-21. Cells were gated on viable CD4^+^ lymphocytes. Intensity of CXCR5 expression on survivin+ CD4 cells is shown **F.** The frequency of CXCR5^+^ cells within survivin^+^ and survivin^−^ CD4 subsets stimulated with αCD3 + IL-12 **G.** Intracellular production of IL-21 within the CXCR5^+^survivin^+^ and CXCR5^+^survivin^−^ CD4 cells stimulated with αCD3 + IL-12 is shown by histogram **H.** Frequency of PD-1^+^ IL-21^+^ cells is shown by box plots **I.** The Wilcoxon matched-pairs signed rank test to compare differences. Boxes and lines represent IQR and median, respectively, and error lines indicate min and max values.

Intracellular expression of survivin was analyzed by flow cytometry in the PBMC of RA patients (*n* = 21) and healthy subjects (*n* = 10) and revealed an enrichment of survivin^+^ cells within the memory CD45RA^−^CD4^+^ T cells compared to naïve (CD45RA^+^) cells in RA patients. In RA patients, the difference was seen both with respect to the propensity (46.0% vs 26.6%, *p* = 0.0012) and to the intensity (MFI: 3654 vs 2256, *p* = 0.007) of survivin expression (Figure 1A, 1B). In healthy controls, survivin+ cells were more prevalent in the naïve compared to memory CD4+T cells (33.4% vs. 56.4%, *p* = 0.041) and had no difference in the intensity of survivin expression (MFI, median: 3666 vs 3633).

The survivin^+^CD4^+^ cells expressed chemokine receptor CXCR5 essential for the GC localization of Tfh cells. Actually, CXCR5 was expressed almost exclusively within survivin^+^ population of CD4^+^ T cells (Figure 1C). Functional Tfh cells require expression of master transcription regulator Bcl-6 [22, 49]. Bcl-6 was identified in 2.5–38% of the survivin^+^ memory CD4^+^ cells, which was more prevalent compared to survivin^−^ memory CD4^+^ cells (Table 1, Figure 1D). Presence of Bcl-6 was associated with higher survivin expression within the survivin^+^CXCR5^+^ cells (Figure 1E).

**Table 1:** Clinical characteristics of patients with rheumatoid arthritis

*n* = 21	Mean ± SD [range]
Survivin^+^ Bcl-6^+^	13.9 ± 10.1 %
(CD45RA^−^CD4^+^)	4040 ± 1393, MFI^a^
Gender, F/M	16/5
Age, years	64 ± 9.6
Disease duration, years	20.4 ± 11.6
RF^b^/ACPA^c^ positive, *n*	20
DAS28^d^	2.4 ± 0.89 [1.0-3.86]
MTX^e^, mg/week	15.8 ± 5.0
Infliximab, g	9.6 ± 4.2 [2.6-16.6]

a Mean fluorescence intensity,

b Rheumatoid factor,

c Anti citrullinated protein antibodies,

d Disease Activity Score in 28 Joints,

e Methotrexate

To verify expression of survivin during Tfh cell formation, human PBMC were forced into Tfh phenotype by CD3 activation combined with IL-12 or IL-21. Adoption of Tfh phenotype was evaluated by expression of CXCR5 receptor and the intracellular production of IL-21 within the PD-1^+^survivin^+^ CD4 cells. Cell stimulation with αCD3 + IL-12 and αCD3 + IL-21 resulted in up-regulation of CXCR5 on the survivin^+^ CD4 cells, which was more prominent with the αCD3 + IL-12 stimulation (Figure 1F). The αCD3 + IL-12 stimulation increased IL-21 production on PD-1^+^CXCR5^+^survivin^+^ cells compared to αCD3 stimulated cell cultures (Figure 1G). Stimulation with αCD3 + IL-12 enlarged the CXCR5^+^PD-1^+^survivin^−^ population of CD4 cells (Figure 1F). This population showed no increase in the IL-21 production (Figure 1G).

### Survivin and Bcl-6 localize within the nuclei of human CXCR5+ T cells

**Figure 2:**
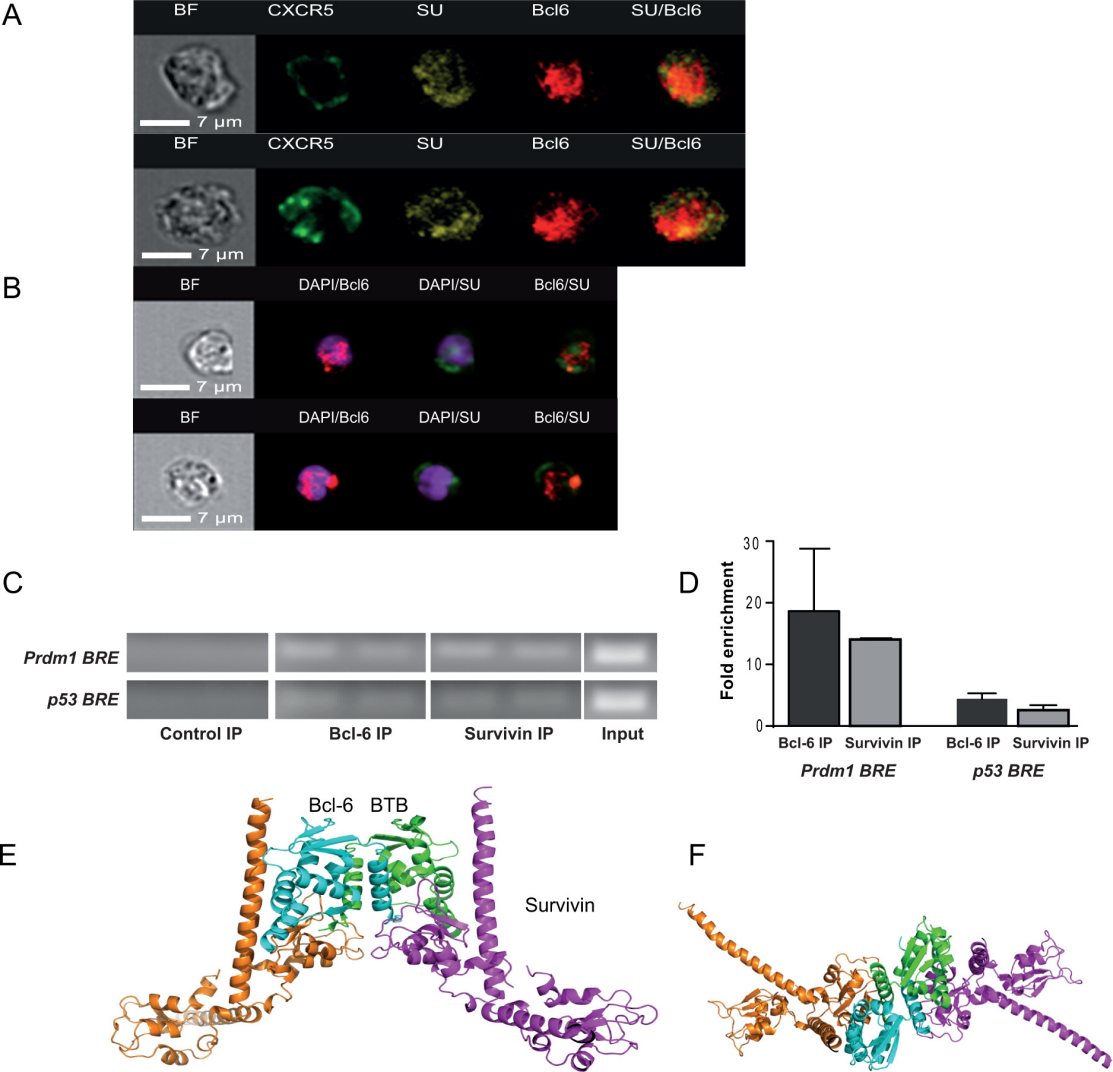
Survivin and Bcl-6 co-localize and interact in human PBMC Freshly isolated human PBMCs were stained with CXCR5, survivin and Bcl-6, and acquired on an imaging flow cytometer. Cells were gated on CXCR5+ lymphocytes. Localization of CXCR5 (green), survivin (yellow) and Bcl-6 (red) within the same cell could be observed. Co-localization of survivin and Bcl-6 is recognized in the right-side column by yellow staining. Left column shows bright field (BF) analysis of the cells **A.** CD4 cells isolated from PBMC were stimulated with concanavalin A (5 μg/ml, 3 h) and stained for Bcl-6 (red) and survivin (green). Nuclear chromatin was stained with DAPI (purple). The nuclear localization of Bcl-6 and survivin is visualized within the cells **B.** DNA of human PBMC (*n* = 4), stimulated with LPS/concanavalin A, was immunoprecipitated (IP) with anti-survivin and anti-Bcl-6 antibodies and used in a ChIP assay. Normal IgG was used as a negative control. The IP DNA was then subjected to PCR using primer sets spanning the Bcl-6 response element (BRE) within the *p53* promoter or the Blimp-1 gene, *Prdm1* **C.** The input refers to PCR using 1% of total chromatin before IP. Quantification of ChIP analysis is presented as fold enrichment to the control IP **D.** A docking model of the putative survivin-Bcl-6 interaction is proposed and viewed perpendicular to **E.** and along **F.** the 2-fold axis of Bcl-6 BTB. The Cα ribbons of the survivin dimers are depicted in magenta (representing the 1st cluster) and orange (representing the 4th cluster), while the docking partners (Bcl-6 BTB subunits) are depicted in cyan and green, respectively.

The co-expression of survivin and CXCR5/Bcl-6 within Tfh cells was further confirmed by imaging flow cytometry analysis. Freshly isolated human PBMC gated on CXCR5^+^ cells comprised a subset that co-expressed both survivin and Bcl-6 (Figure 2A), confirming the above described flow cytometry data. The images reveal CXCR5 position at the periphery of the cells, forming uneven clusters of higher intensity along the plasma membrane. Under the non-stimulated conditions, the staining for survivin was predominantly identified in the cytoplasma, often in the vicinity or within the CXCR5 clusters attached to the cell membrane. Bcl-6 staining was mainly localized to the nucleus of CXCR5^+^ cells. In the non-stimulated cells, only a few of the survivin pixels had nuclear location. In the isolated and activated CD4^+^ T cells, the nuclear co-localization of survivin and Bcl-6 was clearly observed (Figure 2B). Survivin is seen enriched in the perinuclear area and its partial translocation into the nucleus occurred, which corresponded to co-localization of the survivin and DAPI staining. The stimulated T cells had intense and more condensed nuclear staining for Bcl-6, where survivin and Bcl-6 were co-localized to the same pixels.

### Survivin binds to the Bcl-6 responsive elements of Blimp-1 and p53 genes

Functional and biochemical evidence for a direct role for Bcl-6 in transcriptional repression are most studied on the genes encoding Blimp-1 (*Prdm1*) [50, 51] and the cell cycle regulator p53 [52, 53]. Therefore, we examined whether survivin bound to the Bcl-6-responsive elements (BRE) of these genes. To detect DNA-bound survivin within BRE of the *Prdm1* gene we performed a chromatin immunoprecipitation (ChIP) analysis of human LPS/Concanavalin A-stimulated PBMC. The immunoprecipitation with anti-survivin antibodies showed that the amplified BRE was 14–15-fold enriched with survivin in 3 independent experiments (Figure 2C, 2D). The same BRE region showed the 10–30-folds enrichment when immunoprecipitated with anti-Bcl-6 antibodies (Figure 2C, 2D). No enrichment of the BRE region was observed with the isotype-matched control antibodies.

ChIP assays of the promoter region of the *p53* gene, containing BRE, could identify the enrichment of survivin and of Bcl-6 within this region of human LPS/Concanavalin A-stimulated PBMC (Figure 2C, 2D). These results showed that survivin was present on the BRE within the *p53* and *Prdm1* genes in amounts comparable with Bcl-6 itself; therefore, survivin may be required for Bcl-6-dependent repression of these genes.

### A structural model of the survivin-Bcl-6 interaction

Given the amount of evidence supporting the co-localization of survivin with Bcl-6, we next hypothesized a direct interaction between these proteins and how this putative complex may form. Bcl-6 contains a versatile protein-protein interaction motif known as BTB domain, a primary interaction site with its co-repressors [54, 55], at the N-terminus and a typical array of six C2H2 zinc-finger domains connected by an undefined proline-rich region. We uploaded the crystal structure of the unbound human Bcl-6 BTB domain (PDB code 1R28) [55] and the survivin dimer (PDB code 1e31) [56] to the Cluspro2 [57] protein-protein docking server, which excelled when entered into the CAPRI [58, 59] docking prediction competition. Both the “standard” and “antibody” modes were tested, but the most plausible models were derived using the “antibody” mode. The best four clusters contained similar complexes presented in Figure 2E and 2F. In these models, the survivin BIR domain interacts with Bcl-6 through a shallow cleft on the Bcl-6 BTB domain. The C-terminus of survivin binds tightly to the side of the Bcl-6 BTB domain. The helices of the Bcl-6 BTB domain (aa56–62) interact with the C-terminal helix-turn-helix motif of survivin (aa102–125). The predicted model permits the independent interaction of each survivin subunit with a Bcl-6 subunit, potentially forming linear multimers with 2-fold symmetry.

### Inhibition of survivin transcription affects follicular T cells and reduces antibody production in arthritic mice

**Figure 3:**
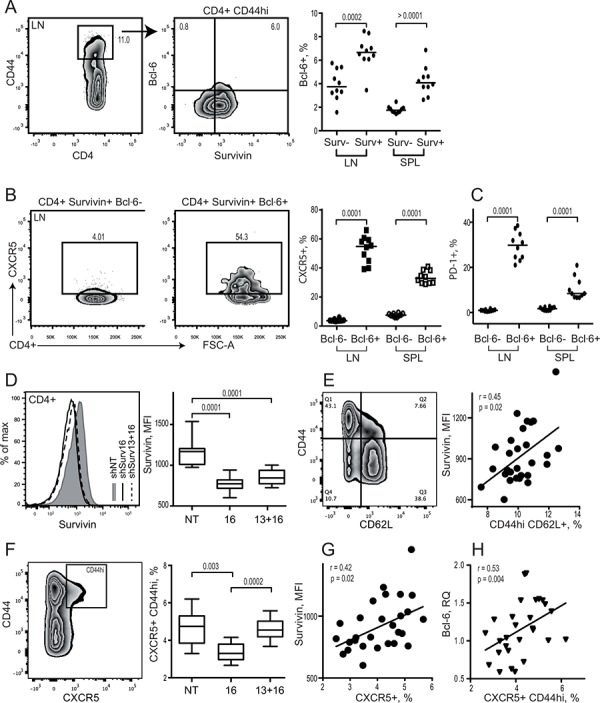
Survivin positive subset of CD44^hi^ CD4 lymphocytes in mouse possess a complete phenotype of Tfh cells Spleen and lymph nodes from collagen II immunized arthritic (CIA) mice were analyzed for expression of survivin and Bcl-6 using flow cytometry **A.** Cells were gated on memory CD44^hi^CD4^+^ lymphocytes. Expression of CXCR5 **B.** and PD-1 **C.** was investigated within Bcl-6^+^ survivin^+^ and Bcl-6^−^ survivin^+^ cells. Dots represent individual mice and the horizontal line shows median of the group. Survivin translation in CIA mice was inhibited by shRNA-producing constructs provided as a single intra-peritoneal injection (shSurv16, *n* = 10, or shSurv13+16, *n* = 10). Control mice were treated with a non-targeting RNA construct (shNT, *n* = 9). Survivin expression in spleen was analyzed by flow cytometry 12 days after the injection. Cells were gated on CD4^+^ lymphocytes. Intensity of survivin expression (MFI) within the groups is shown by a representative histogram and summarized in a box plot **D.** Survivin expression (MFI) on CD4 lymphocytes correlated to the size of CD44^hi^CD62L^+^ population **E.** Expression of CXCR5 on CD44^hi^CD4+ lymphocytes in the groups is shown as box plot **F.** CXCR5^+^ population correlates with the intensity of survivin **G.** and with Bcl-6 mRNA **H.** Boxes and lines represent IQR and median, respectively, and error lines indicate min and max values. The Mann-Whitney *U*-test was used to compare differences between groups. Correlation analyses were performed using Spearman's test.

We first studied if expression of survivin is a part of Tfh phenotype in mouse spleen and lymph nodes (LN) by flow cytometry. Similar to human PBMC, survivin was expressed in memory CD44^hi^CD4^+^ cells of LN and spleen in mice (Figure 3A). These survivin^+^ cells were recognized by high expression of Bcl-6. The majority of survivin^+^Bcl-6^+^ cells in the LN and spleen resided within the CXCR5^+^ population (Figure 3B). The survivin^+^CXCR5^+^ CD4 cells were also recognized by high expression of PD-1 receptor (Figure 3C), indicating that survivin is a characteristic feature of mouse Tfh cells in spleen and LN.

We next examined if survivin is required for formation of Tfh cells in arthritic mice. Inhibition of survivin by shRNA-producing lentiviral constructs (shSurv) induced a profound reduction in the intensity of survivin in CD4^+^ cells (Figure 3D) and in the number of survivin^+^ cells in the spleen [47]. The decrease of survivin intensity was directly related to the reduction of memory CD44^hi^CD62L^+^ CD4 cells in the shSurv treated mice (Figure 3E). shSurv treatment resulted in a reduced CXCR5^+^ subset within the CD44^hi^CD4^+^ cells (Figure 3F), which correlated with the decreased survivin intensity (Figure 3G).

**Figure 4:**
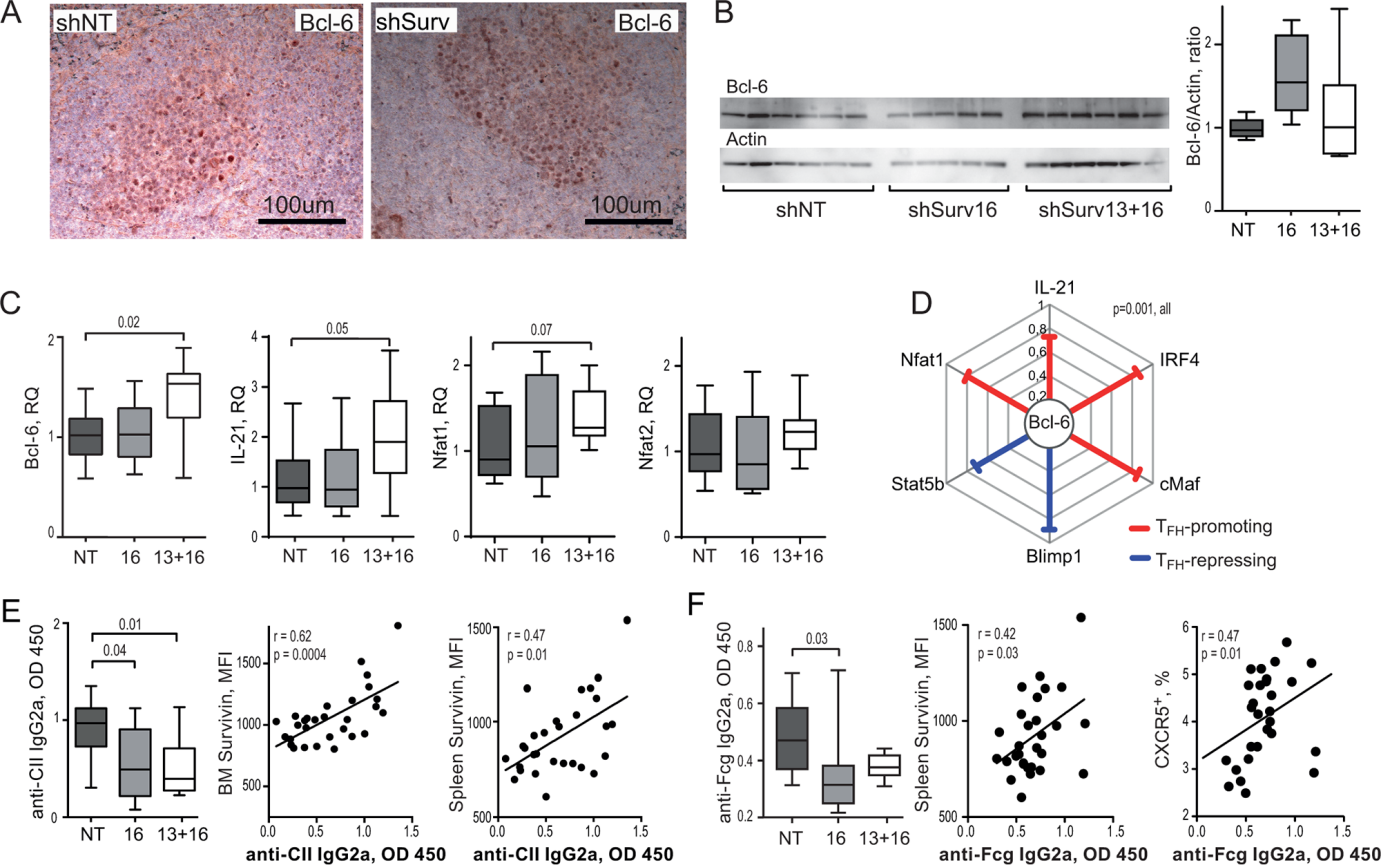
Inhibition of survivin disturbs Bcl-6 dependent mechanisms of antibody production in experimental arthritis Bcl-6 expression was analyzed in CIA mice treated with the survivin inhibiting (shSurv16 and shSurv13+16) and control (shNT) shRNA constructs, by immunohistological staining for Bcl-6 in spleen **A.** and by Western blot on bone marrow cells **B.** Bcl-6 was quantified in ratio to actin of each sample. Statistics for the groups is presented as box plots. Transcription of Bcl-6, IL-21, Nfat1 and Nfat2 in spleen was analyzed by RT-PCR and presented in relative quantity (RQ) to the median of the control group **C.** Spearman's rank correlation coefficient (rho) between the transcription of Bcl-6 vs. Blimp-1, Stat5b, Nfat1, IL-21, IRF4, and cMaf in spleen of CIA mice are shown **D.** Anti-collagen II (anti-CII) antibodies **E.** and anti-Fcgamma (anti-Fcg) antibodies **F.** in the serum of CIA mice treated with shSurv or shNT were measured by ELISA. Anti-CII antibodies correlated with survivin intensity in bone marrow and in spleen, measured by flow cytometry **E.** Anti-Fcg antibodies correlated with the intensity of survivin and with size of CXCR5^+^ CD4 population in spleen, measured by flow cytometry **F.** Boxes and lines represent IQR and median, respectively, and error lines indicate min and max values. The Mann-Whitney *U*-test was used to compare differences between groups. Correlation analyses were performed using Spearman's test.

Bcl-6 controls the maturation and function of Tfh cells and GC formation by inducing expression of the homing receptor CXCR5 [60, 61]. Bcl-6 mRNA in the spleen of shSurv treated mice was directly related to the size of CXCR5^+^CD44^hi^ population of CD4 T cells (Figure 3H). shSurv treated mice formed sufficient Bcl-6^+^ areas within the spleen follicles which were comparable to the controls (Figure 4A). The protein content of Bcl-6 in shSurv treated mice was similar to the controls (Figure 4B) despite the increase in Bcl-6 mRNA, and also in IL-21 mRNA promoting Bcl-6 transcription (Figure 4C). The mRNA levels of Bcl-6 correlated positively to the promoters of its transcription (IL-21, IRF4, c-Maf, NFAT1) and to Blimp-1 and Stat5 repressing Bcl-6 transcription (Figure 4D). This could reflect the combined input of these mechanisms in regulation of Bcl-6 in the shSurv treated mice. Transcription factor NFAT2 is important for the expression of CXCR5 in Tfh cells acting independently of Bcl-6 [62]. The shSurv treated mice had no significant change in NFAT1 and NFAT2 mRNA levels (Figure 4C).

Consistent with insufficiency in Tfh cells, shSurv-treated mice immunized with CII had decreased production of antigen-specific and pathogenic anti-CII IgG_2a_ antibodies (Figure 4E) and also of anti-Fc-gamma IgG_2a_ autoantibodies (RF) compared to controls (Figure 4F). Anti-CII IgG_2a_ levels correlated with the reduced survivin^+^ population in the bone marrow (*r* = 0.62, *p* = 0.0004) and spleen (*r* = 0.47, *p* = 0.01) (Figure 4E). The levels of RF showed correlation to the intensity of survivin expression in spleen cells and with the reduced size of CXCR5^+^CD4^+^ subset (Figure 4F).

### Immunization with survivin-derived peptides restrained Tfh population and enlarged autoantibody production

**Figure 5:**
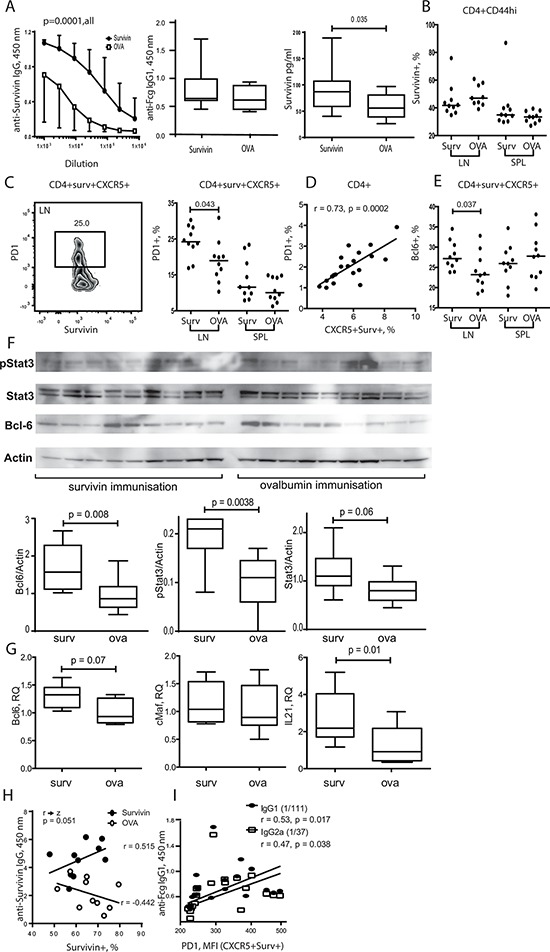
Exposure to survivin enriched PD-1^+^ Bcl-6^+^ subset of Tfh cells via STAT3 dependent mechanisms DBA/1 mice were immunized with survivin-derived peptide (100 μg/mouse × 4, subcutaneously). Control mice were immunized with ovalbumin-derived peptide (OVA). Both groups were then subjected to collagen-induced arthritis. Anti-survivin IgG antibodies, anti-Fcg antibodies, and survivin levels in serum were measured by ELISA **A.** Flow cytometry analysis of the expression of survivin **B.** in CD44^hi^CD4^+^ lymphocytes, and expression of PD-1 **C.** and Bcl-6 **E.** in survivin^+^CXCR5^+^ CD4^+^ lymphocytes in spleen (SPL) and lymph nodes (LN). PD-1 expression correlated to the size of CXCR5^+^survivin^+^ population **D.** Dots represent individual mice and the horizontal line shows median of the group. Protein levels of active STAT3 phosphorylated at Y705 (pStat3), total Stat3, Bcl-6 and actin in spleen were analyzed by the Western blot **F.** The levels of each protein were quantified in ratio to actin of each sample. Quantification of the detected bands is presented as box plots. Transcription of Bcl-6, cMaf and IL-21 in the spleen was analyzed by RT-PCR and presented in relative quantity (RQ) to the median of the control group **G.** Anti-survivin antibodies in serum correlated with the size of survivin^+^ CD4 population in spleen of survivin-immunized mice **H.** Comparison of the correlations in the survivin- and OVA-immunized groups is done by the Fisher r-to-z transformation analysis. Anti-Fcg antibodies correlated with the intensity of PD-1 on the CXCR5^+^survivin^+^ CD4 lymphocytes **I.**, as measured by flow cytometry. Box plots with line represent IQR of the group and median, respectively, and error lines indicate min and max values. The Mann-Whitney *U*-test was used to compare differences between groups. Correlation analyses were performed using Spearman's test.

Since high serum levels of survivin are prevalent in RA patients [40, 63], we ask if exposure to survivin peptides by immunization increases Tfh subset in mice. Arthritis prone DBA/1 mice were immunized with survivin-derived peptide and developed high levels of anti-survivin antibodies (Figure 5A) indicating a sufficient immunization response. Survivin-immunized mice had also increased production of anti-Fc-gamma IgG_1_ autoantibodies and serum survivin (Figure 5A) compared to OVA-immunized control mice, which resembled the phenotype of RA patients.

The total survivin^+^ population within the mature CD44^hi^CD4^+^ cells (Figure 5B) and CXCR5^+^CD4^+^ cells (not shown) of the survivin-immunized mice was similar with the controls. Survivin-immunized mice had significantly increased population of PD-1^+^ (Figure 5C) and Bcl-6^+^ cells (Figure 5E) within survivin^+^CXCR5^+^ CD4 cells in LN, suggesting enhanced maturation and activity of Tfh cells in the survivin-immunized mice. PD-1 expression on CD4 cells was in direct proportion to the size of survivin^+^CXCR5^+^ population (*r* = 0.73, *p* = 0.0002) (Figure 5D).

The survivin-immunized mice had higher production of Bcl-6 protein in spleen tissue compared to control OVA-immunized mice (Figure 5F). Transcriptional regulation of Bcl-6 relies on activation of transcription factor STAT3 [8]. The survivin immunized mice displayed a significant increase of the levels of total and Tyr705 phosphorylated STAT3 in western blot analysis of spleen cells (Figure 5F), which could be responsible for the increased transcription of Bcl-6 and IL-21 observed in the survivin-immunized mice (Figure 5G).

Consistent with the increased activity of Tfh cells, survivin-immunized mice had significantly increased level of anti-survivin antibodies (Figure 5A), which was also correlated with Bcl-6^+^ Survivin^+^ CXCR5^+^ subset of CD4 cells (*r* = 0.44, *p* = 0.05). Notably, the levels of anti-survivin antibodies appeared to be in direct dependence of the survivin^+^ CD4 subset, and this reliance was not present in the OVA-immunized controls (Figure 5H). The production of anti-Fc-gamma autoantibodies was readily detected in survivin-immunized mice (Figure 5B). The intensity of PD-1 expression on survivin^+^CXCR5^+^ CD4 cells was in direct proportion to the level of anti-Fc-gamma antibodies (Figure 5I).

## DISCUSSION

This study demonstrates that intracellular expression of the oncoprotein survivin is a characteristic feature of Tfh cells in RA patients and in arthritic mice. The survivin positive subset of memory CD4 T cells possessed a complete signature of mature Tfh cells expressing CXCR5 and PD-1 receptors and master transcription factor Bcl-6 required for Tfh function [1, 22]. We also show that survivin expression is essential for formation and function of Tfh cells. Human survivin^+^ CD4 cells stimulated with IL-12 adopt Tfh phenotype *in vitro* as evidenced by the increased expression of CXCR5 on survivin^+^ CD4 cells. This CXCR5^+^ survivin^+^ subset is shown responsible for the increased IL-21 production. This link between IL-12 and survivin-dependent maturation of Tfh cells deserves attention due to its replication in pre-symptomatic RA individuals where a strong association of serum survivin with IL-12 is found [48].

Taking advantage of the experimental arthritis model, we demonstrated that inhibition of survivin resulted in reduction of Tfh cells in the shSurv treated mice. This occurs through a limitation of the surface expression of CXCR5, which controls trafficking of Tfh cells into follicles and communication with B cells [64, 65]. This limitation of CXCR5 expression on Tfh cells was clinically significant since it was associated with a decreased production of pathogenic anti-CII antibodies and confirmed the key role of survivin for Tfh maturation and function. In consistence with the pathogenic part of anti-CII antibodies in arthritis, the reduction of survivin expression delayed arthritis development and alleviated its severity [47].

Expression of CXCR5 is governed by the transcriptional repressor Bcl-6. Overexpression of Bcl-6 enhances CXCR5 and PD-1 through a micro-RNA dependent mechanism [66], while Bcl-6 deficient mice obtained by germline deletion [6] or by selective conditional approach in CD4 cells [67] had a reduced number and higher rate of apoptosis in CXCR5^+^ PD-1^hi^ Tfh cells. Transcription of Bcl-6 was not inhibited by survivin deficiency, and shSurv treated mice had an increase of Bcl-6 mRNA. Notably, survivin inhibition had no effect on the transcriptional regulators of Th1, Th2 and Treg lineages, which is in line with a distinct place of survivin in maturation of Tfh cells. Bcl-6 is known to repress activity of the genes controlling genomic stability [49, 68]. It protects malignant and damaged cells from apoptosis enabling cell cycle and may sustain proliferation of autoreactive T and B cell clones. Bcl-6 activity may predispose to aberrant immunity and permit production of autoantibodies. Rheumatoid factor, anti-Fc-gamma antibodies, belongs to the canonical autoantibody repertoire in RA. Production of these autoantibodies is detected in the experimental arthritis studied herein. Consistent with Bcl-6 dysfunction, the inhibition of survivin significantly diminished autoantibody production.

Bcl-6 is known to repress transcription of the Blimp-1 gene [7]. A release of Blimp-1 expression together with limited production of CXCR5 in shSurv treated mice exemplified insufficient function of Bcl-6 under survivin-deficient conditions. This resulted in ascending Blimp-1 control of lymphocyte maturation through inhibition of IL-2 and promoting memory CD8 T cells [47]. Imaging analysis of human CD4 cells shows co-localization of survivin with Bcl-6 within the nuclei of activated CXCR5^+^ cells. Its co-precipitation with the BRE in the promoter region of the Blimp-1 gene further supports functional synergy between survivin and Bcl-6. Our findings are in line with recent reports on the ability of survivin to bind chromatin [69, 70], and to regulate transcriptional activity of target genes [71]. The proposed structural model illustrates potential interaction between the BIR domain of survivin and the BTB domain of Bcl-6, and predicts survivin monomers to cross-link the Bcl-6 BTB dimers resulting in the oligomerization and activation of Bcl-6. This hypothetic model of survivin binding resembles those described for histone H3 and Smac/DIABLO [72, 73] and requires further prove in experimental structural studies.

Immunization experiments using survivin-derived peptides demonstrates that exposure of Tfh cell to extracellular survivin, clinically present in a substantial part of RA patients [63], serves as external regulator of Tfh cells. Survivin immunization induced no significant enlargement of Tfh subset, where the partiality of CXCR5^+^ Survivin^+^ cells was similar to controls. It rather enhanced expression of PD-1 and Bcl-6 on the existing CXCR5^+^ survivin^+^ Tfh cells in LN restraining their activity. We show that the survivin immunized mice have increased production and activation of STAT3, which mediates pleiotropic cytokine signaling in RA [74]. In the Tfh cells of the survivin immunized mice, STAT3 activation together with the increased transcription of IL-21 results in the accumulation of PD-1^+^ Bcl-6^+^ phenotype. [75, 76]Additionally, activation of STAT3 provides a positive feedback loop of survivin production by direct binding and activation of the survivin gene [77].

Taken together, the present study adds information on the phenotype and functional characteristics of Tfh cells in autoimmunity. We show that onco-protein survivin is required to induce and to maintain Tfh cell development and function. Survivin controls the CXCR5 expression and IL-21 production by CD4 lymphocytes coordinating activity of Bcl-6, the key regulator of Tfh maturation. Inhibition of survivin reduced formation of Tfh cells and autoantibody production. Extracellular exposure to survivin enhances Bcl-6 and PD-1 expression on Tfh cells in STAT3-dependent manner. These findings highlight the important role of the survivin─Bcl-6 axis in the pathogenesis of RA and provide a new link between transcriptional regulation of T cells and autoimmunity.

## MATERIALS AND METHODS

### Patients and sample collection

Peripheral blood (PB) samples were collected from 21 patients with established RA, diagnosed according to the ACR 1987 criteria [78]. All patients were recruited from the Rheumatology Clinic at the Sahlgrenska University Hospital, Gothenburg, Sweden, during the period from January 2014 to September 2014, and all patients gave written informed consent to the study. Clinical and demographic characteristics of the patients and their immunosuppressive treatment are presented in Table 1. The study is approved by the Regional Ethics Board in Gothenburg, Sweden (Dnr 633–07).

### Collagen-induced arthritis

H-2q restricted DBA/1 mice (8 weeks old, male, Taconic Europe A/S, Ry, Denmark) were immunized with collagen type II (CII, 100 mg/mouse) emulsified in an equal volume of complete (day 0, intradermally) and incomplete (day 21, subcutaneously) Freund's adjuvant (Sigma-Aldrich, St. Louis, MO, USA) as described [79, 80]. Disease progression was followed by assessment of weight and signs of arthritis in the individual mice. Samples of serum, bone marrow, spleen, draining lymph nodes and joints were collected for further analysis. All mice were housed at the animal facility at the Department of Rheumatology & Inflammation Research, under standard conditions of temperature and light, and fed laboratory chow and water *ad libitum*. The experimental setting is approved by the animal ethics committee at the University of Gothenburg (permits 319–2011 and 272–2010).

### Inhibition of survivin *in vivo*

Lentiviral construct, MISSION^®^ TRCN0000054613 (shSurv13) and TRCN0000054616 (shSurv16) (Sigma-Aldrich), encoding small hairpin(sh)RNA targeting survivin, were used. Both shRNA target all three known isoforms of mouse survivin. The non-targeting shRNA controls (shNT) were subjected to MISSION^®^ pLKO.1-puro non-mammalian shRNA control transduction particles (SHC002H) (Sigma-Aldrich). The constructs were given by a single intra-peritoneal injection (2-6 × 10^6^ transduction particles in 100 μl/mouse) at the signs of clinical arthritis. Experiments were terminated 12 days after the injection of constructs. The toxicity of shRNA lentiviral particles has been investigated earlier [47].

### Immunization with survivin-derived peptides

DBA/1 mice were immunized with a 15aa survivin peptide (SVN53–67/M57-KLH) predicted to bind MHC class I and MHC class II alleles [81]. Control group was immunized with 15aa ovalbumin peptide (OVA-KLH). The 100 μg of peptides were emulsified with the incomplete Freund's adjuvant and delivered subcutaneously once weekly on 4 occasions, beginning 10 weeks ahead of the immunization with collagen II.

### Cell isolation and stimulation

PBMC were isolated by density gradient separation on Lymphoprep (Axis-Shield PoC As, Norway). Cells were washed with PBS and cultured 1 × 10^6^/ml in RPMI medium (RPMI1640, Gibco) supplemented with 5% FCS, 2 mM Glutamax-1 (Gibco), 50 μM β_2_-mercaptoethanol and 50 μg/ml gentamicin (Sanofi). To facilitate formation of Tfh cells, PBMC were activated with anti-CD3 antibodies (0.25 μg/ml, SAB4700041, Sigma-Aldrich) alone or in combination with IL-12 (20 ng/ml, BioSite, Sweden) and IL-21 (50 ng/ml, BioSite) for 5 days. GolgiPlug^−^ (555029, BD Biosciences) was added for the last 12 h of incubation to enable intracellular enrichment of IL-21. For imaging flow cytometry, CD4 T cells were isolated using positive magnetic beads separation followed by the bead detachment process as described (CD4^+^ isolation kit, InVitrogen). The isolated CD4 cells were stimulated with Concanavalin A (5 μg/ml, Sigma-Aldrich) for 3 h.

Bone marrow cells from femur and tibia of arthritic mice were flushed with PBS. Spleens and lymph nodes were mashed through a 70 μm cell strainer. Erythrocytes were lysed in NH_4_Cl solution (0.83%, pH 7.29), and cell pellets were washed with PBS and re-suspended in FACS buffer (PBS, 10% FBS, 0.09% NaN3, 0.5 mM EDTA). Cells for RNA preparation were lysed in RLT buffer (Qiagen, Valencia, CA, USA). Cells for western blot were lysed and sonicated in lysis buffer containing protease and phosphatase inhibitors (Complete Mini and PhosSTOP; Roche Diagnostics GmbH, Basel, Switzerland).

### Flow cytometry

Single cell suspensions in FACS buffer (PBS, 10% FBS, 0.09% NaN3, 0.5 mM EDTA) were pre-incubated with Fc-block (BD Bioscience) as described [82]. For staining of human cells following antibodies were used: Anti-CD4 (SK3), anti-CXCR5 (RF8B2), anti-PD-1 (EH12.1) and anti-IL-21 (3A3-N2.1), purchased from BD Biosciences; anti-CD19 (H1B19), anti-CD45RA (HI100), from BioLegend. For staining of mouse cells the following antibodies were used: anti-CD4 (RM4–5, BD Biosciences); CD44 (IM7), anti-CXCR5 (SPRCL5), anti-PD-1 (RMPI-30) and CD62L (MEL-14) from eBioscience. Intracellular staining for Bcl-6, survivin and IL-21 was performed as previously described and according to the manufacturer's protocol [37]. Anti-Survivin (91630) and isotype control (mouse IgG1κ) were purchased from R&D Systems, and anti-Bcl-6 (K112–91), isotype control (mouse IgG1κ, MOPC-21) were purchased from BD Biosciences and used for both human and mouse cells.

Stained cells were collected (5 × 10^5^ events/sample) by a FACSCanto II with FACSDiva software (BD Biosciences), and data evaluated using the FlowJo software (version 10.0.6, Tree Star Inc., Ashland, OR). Compensation for fluorochrome interferences was done using single stained CompBeads (BD Bioscience). Gating of the cells was based on the isotype control or on the fluorochrome minus one setting. Data is shown as percentage or mean fluorescence intensity (MFI), calculated in FlowJo and compared to data acquired in the same run.

### Imaging flow cytometry

Cells were stained with FITC-conjugated anti-CXCR5, PE-conjugated anti-survivin, and Alexa Fluor 647-conjugated anti-Bcl-6 antibodies, with the addition of DAPI (3 μM) to visualize cell nuclei. Samples (1 × 10^5^ cells) were acquired on an imaging flow cytometer (ImageStreamX MkII, Amnis), and analysed using IDEAS v. 6.1 software (Amnis). Briefly, following colour compensation, focused, single cells were gated. Co-expression of CXCR5, survivin and Bcl-6 was then analysed in this population using the intensity feature in IDEAS.

### Gene expression analysis

Total RNA from splenocytes was extracted using an RNeasy Mini Kit (Qiagen, Valencia, CA). The concentration and quality of the RNA were evaluated with a NanoDrop spectrophotometer (Thermo Scientific, USA) and Experion (Bio-Rad laboratories Inc., USA). Real-time amplification was performed with RT^2^ SYBR^®^ Green qPCR Mastermix (Qiagen) using a ViiA™ 7 Real-Time PCR System (Applied Biosystems) as previously described [83]. The level of mRNA for Bcl-6, IL-21, NFAT1 and NFAT2 [62], Stat5b, IRF4, cMaf and Blimp-1 was measured. Expression levels of these genes were normalised to those of two reference genes, *Gapdh* and *Ppia* (TATAA Biocenter, Sweden). The sequences of primers used in the real-time PCR reaction are available upon request. The results were expressed as the fold change compared with the expression levels in the control cells with the ddCq-method.

### Protein preparation and western blotting

Protein concentrations in cell lysates were measured by use of the Bicinchoninic Acid Protein Assay kit (Pierce, Rockford, IL, USA), according to the manufacturer's protocol. Proteins were separated on SDS-PAGE, 4–12% Bis-Tris gels (NuPAGE; Invitrogen), and transferred to polyvinylidene difluoride membranes (NuPAGE; Invitrogen), which were blocked with 5% BSA and incubated with anti-pStat3-Y705 (ab76315; Abcam, Cambridge, United Kingdom), anti-Stat3 (#4904; Cell Signaling, Danvers, MA), anti-Bcl-6 (sc858; Santa Cruz Biotechnology, Santa Cruz, CA), or rabbit antiactin antibodies (A2066; Sigma-Aldrich) at 4°C overnight. Detection was performed with peroxidase-conjugated anti-rabbit secondary antibody (NA934VS; GE Healthcare Life Sciences, Pittsburgh, PA, USA) and Amersham ECL Select substrate (GE Healthcare Life Sciences). Chemiluminescent signals were visualized and quantified by the ChemiDoc equipment and Quantity One software (Bio-Rad Laboratories, Hercules, CA, USA).

### Autoantibody measurements

Antigen specific antibodies to collagen type II, and antibodies to Fc-gamma (RF) were measured in serum using ELISA as described previously [80, 84]. Measurements were performed using a serial dilution of serum (1:10^2^–1:10^7^) and results are expressed as the absorption at 450 nm.

### Chromatin immunoprecipitation (ChIP) analysis

Human PBMC were isolated by density gradient separation on Lymphoprep (Axis-Shield PoC As, Norway), and cultured in the presence of Concanavalin A (0.625 μg/ml, Sigma-Aldrich) and LPS (5 μg/ml, Sigma-Aldrich) for 72 h. Cells were then cross-linked and lysed according to EpiTect ChIP OneDay kit (Qiagen). After sonication to shear the chromatin, cellular debris was removed by pelleting. After pre-clearing the chromatin, 1% of the sample was removed as “input fraction”. The rest of the sample was incubated with either 2 μg anti-Bcl-6 [85, 86] (N3, Santa Cruz Biotechnology), or anti-Survivin [87] (10811, Santa Cruz Biotechnology). In each experiment, one sample with unspecific antibody was included as negative control for nonspecific binding. A known Bcl-6-targeted sequence within the first non-coding exon of the *Bcl-6* gene was amplified and used as an internal positive control. The immune complexes were washed, the cross-links reversed and the DNA purified according to the EpiTect ChIP OneDay kit (Qiagen). The purified DNA was used as template in real-time amplification using different oligonucleotide pairs for p53 [52], Bcl-6 [86] and Blimp-1 [50]. PCR products were resolved on 2% agarose gels and visualized by ethidium bromide staining and quantified by the ChemiDoc equipment and Quantity One software (Bio-Rad Laboratories).

### Statistical analysis

The median and interquartile range (IQR) for each group was calculated. Comparisons were done by the Mann-Whitney *U*-test or by the Wilcoxon matched-pairs signed rank test. A probability of 0.05 (2 tailed) was used as a significance threshold. Correlation analyses were performed using Spearman's test. Standard software GraphPad Prism (version 6.0 for Mac; GraphPad Software, San Diego, CA, USA) was used for statistical analyses.
